# A case of radical resection for brain metastases of pancreatic cancer after curative chemotherapy for para-aortic lymph node metastases

**DOI:** 10.1186/s40792-022-01461-2

**Published:** 2022-06-06

**Authors:** Takeshi Utsunomiya, Naotake Funamizu, Erina Ozaki, Kei Tamura, Katsunori Sakamoto, Kohei Ogawa, Kosuke Kusakabe, Satoshi Suehiro, Daisuke Yamashita, Mie Kurata, Riko Kitazawa, Yasutsugu Takada

**Affiliations:** 1 Department of Hepato-Biliary-Pancreatic and Breast Surgery, Ehime University Graduate School of Medicine, 454 Shitsukawa, Toon, Ehime 791-0295 Japan; 2 Division of Medical Genetics, Ehime University Hospital, 454 Shitsukawa, Toon, Ehime 791-0295 Japan; 3 Department of Neurosurgery, Ehime University Graduate School of Medicine, 454 Shitsukawa, Toon, Ehime 791-0295 Japan; 4 Department of Pathology, Graduate School of Medicine, Ehime University Proteo-Science Center, 454 Shitsukawa, Toon, Ehime 791-0295 Japan; 5 Division of Diagnostic Pathology, Ehime University Hospital, 454 Shitsukawa, Toon, Ehime 791-0295 Japan

**Keywords:** Metastatic brain tumor, Pancreatic cancer, Treatment

## Abstract

**Background:**

The incidence of brain metastasis of pancreatic cancer has been reported to be approximately 0.3%. The blood–brain barrier of the central nervous system restricts the transfer of substances, including chemotherapeutic agents, from the bloodstream. It is hypothesized that brain metastasis may occur despite successful chemotherapy for the primary tumor. Herein, we report a case of brain metastases of pancreatic cancer that occurred after chemotherapy and discuss relevant literature.

**Case presentation:**

A 64-year-old man underwent distal pancreatectomy with D2 lymph node dissection for resectable pancreatic tail cancer. Invasive ductal carcinoma of pancreas, pT3N2M0 pStageIII (TNM Classification of Malignant Tumors, UICC 8th edition) was diagnosed. S-1 adjuvant chemotherapy was initiated. Three months postoperatively, CA19-9 had increased to 619 U/mL. Additionally, contrast-enhanced computed tomography (CT) and fluorodeoxyglucose-positron emission tomography (FDG-PET)/CT revealed local recurrence in the para-aortic lymph nodes. Chemotherapy was revised to a combined regimen of gemcitabine and nab-paclitaxel. After 4 cycles, tumor markers were normalized. After 5 cycles, recurrence could not be identified on contrast-enhanced CT; therefore, the patient was adjudged to be in complete remission. However, after 29 cycles of chemotherapy, the patient had symptoms of raised intracranial pressure. Magnetic resonance imaging showed two metastatic lesions of 20 mm and 32 mm in the left frontal lobe and cerebellum, respectively. Quasi-emergency resection of the metastatic brain tumors was performed. Pathological examination revealed that the resected specimens originated from primary pancreatic cancer. The patient was discharged on postoperative day 12, without any complications. Postoperatively, a total of 53 Gy of local brain radiation therapy was added. On postoperative day 30, blood carcinoembryonic antigen level had decreased to 5.4 ng/dl and all other tumor markers were negative. Additionally, tumor markers of the cerebrospinal fluid were markedly reduced and the cytology was negative for tumor cells. These results suggested complete resection of the metastatic brain tumors.

**Conclusions:**

Aggressive resection and salvage stereotactic radiotherapy for metastatic brain tumors may lead to complete cure and a good long-term prognosis.

## Background

Brain metastases of pancreatic cancer is rare, with a reported incidence of 0.33–0.6% [1, 2] and cases of resection of brain metastases of pancreatic cancer are extremely rare. Resection of oligo-metastases is a possible treatment option in cases of controlled lesions at other locations. Multiple brain or other metastases are often inoperable with a very poor prognosis [3]. In the present case, recurrence in the para-aortic lymph nodes was controlled with chemotherapy. However, 29 months postoperatively, two metastatic lesions had developed in the brain. The patient underwent radical resection to reduce the symptoms of brain hypertension. Based on postoperative tumor markers and imaging evaluation, complete resection of the tumor was confirmed; the patient has had no recurrence 6 months postoperatively.

## Case presentation

**Fig. 1:**
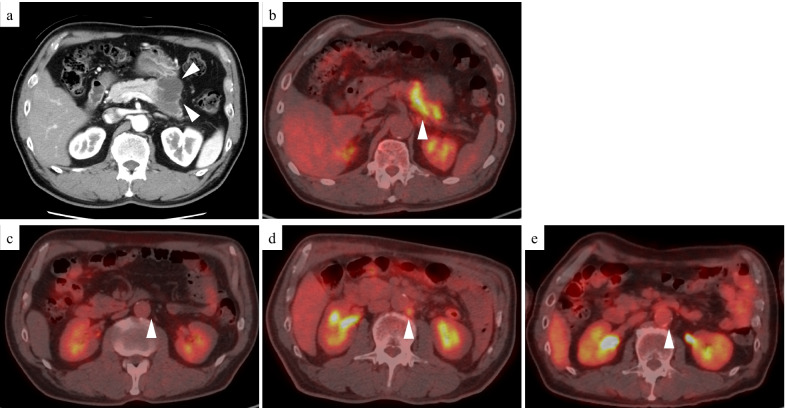
Enhanced computed tomography (CT) and fluorodeoxyglucose-positron emission tomography (FDG-PET)/CT. **a** Enhanced CT horizontal image (arterial phase) shows a pancreatic tail tumor with cystic lesion 46 mm in diameter (arrowhead). **b** FDG-PET/CT image taken before the first surgery: FDG accumulation with a standardized uptake value (SUV)max of 7.1 is observed in the pancreatic tail tumor. **c** FDG-PET/CT image taken before the first surgery: FDG accumulation is not observed in the para-aortic lymph node. **d** FDG-PET/CT image taken before revised chemotherapy regimen: FDG accumulation with a SUVmax of 3.0 is observed in the para-aortic lymph node. **e** FDG-PET/CT image taken after resection of the metastatic brain tumors: FDG accumulation was not observed in the para-aortic lymph node

**Fig. 2:**
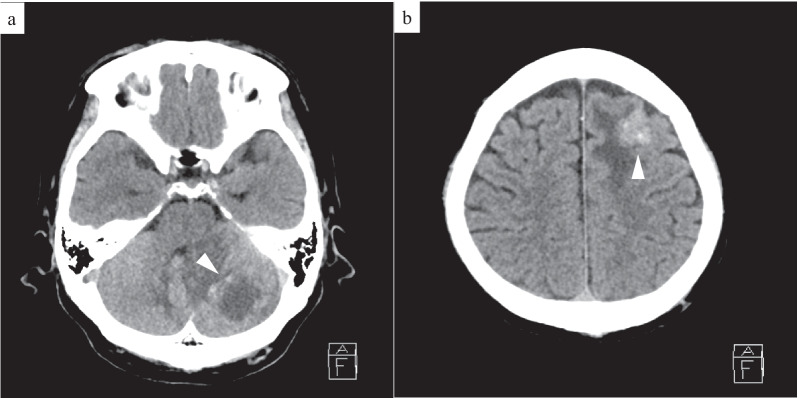
Plain computed tomography (CT) of head. **a** Horizontal CT image: a left cerebellar hemisphere mass with surrounding edema is present (arrowhead). **b** Horizontal CT image: a left frontal lobe mass with surrounding edema is present (arrowhead)

**Fig. 3:**
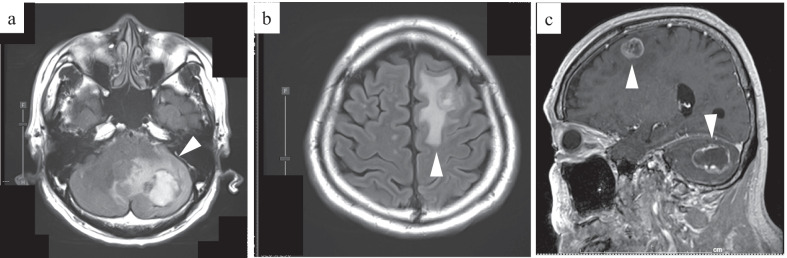
Enhanced magnetic resonance imaging of head. **a** T2-weighted image: A left cerebellar hemisphere mass with extensive surrounding edema is present. **b** T2-weighted image: A left frontal lobe mass with extensive surrounding edema is present. **c** T1-weighted image: The tumor has a contrast effect on the margins and is suspected to be brain metastasis

**Fig. 4:**
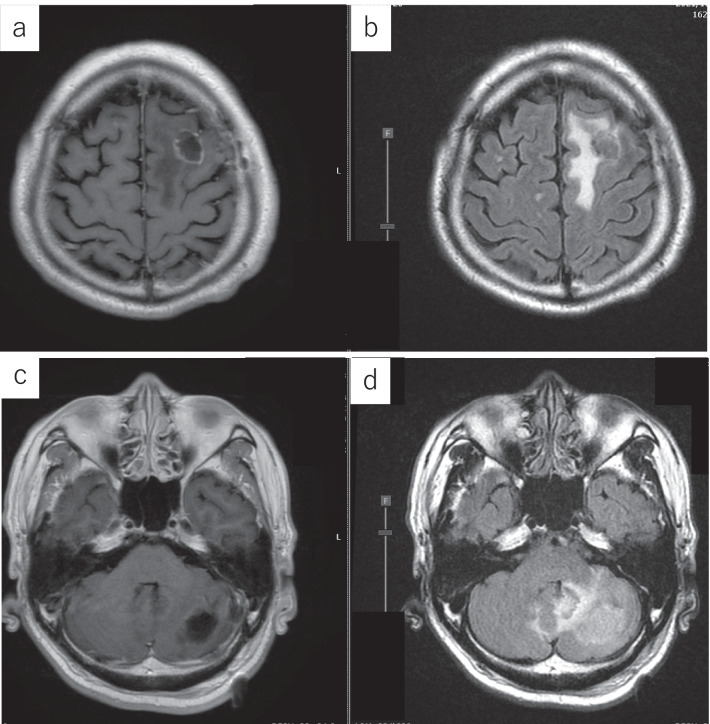
Magnetic resonance imaging after tumor resection. **a**, **c**: T1-weighted image: The tumor is removed. **b**, **d** T2-weighted image: edema around the resection area has improved

**Fig. 5:**
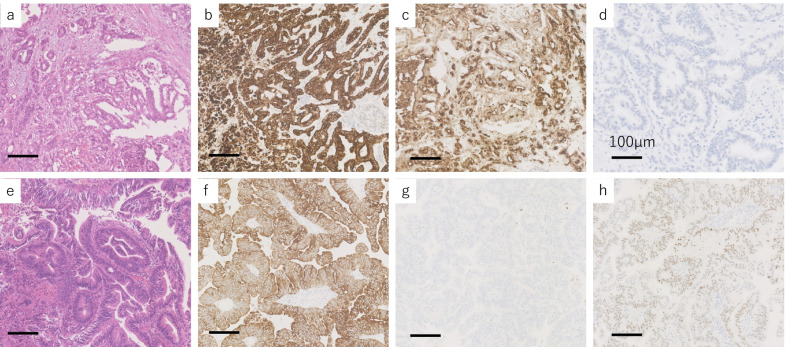
Pathological examinations of primary and metastatic tumors. Histopathological appearance of pancreatic ductal carcinoma (**a**–**d**) and metastatic brain tumor (**e**–**h**). Pancreatic tumor shows duct-like glandular structures (**a**). Metastatic brain tumor resembles intestinal columnar cells (**e**). Both tumors are CK19 positive (**b**, **f**). Pancreatic cancer is MUC1 positive; metastatic brain tumor is negative (**c**, **g**). Metastatic brain tumor is CDX2 positive; pancreatic cancer is negative (**d**, **h**)

A 64-year-old man presented to the hospital with a complaint of left-sided abdominal discomfort. Computed tomography (CT) revealed a cystic lesion of approximately 46 mm in the pancreatic tail (Fig. 1a); additionally, positron emission tomography (PET)–CT showed standardized uptake value, SUVmax: 7.1 of fluorodeoxyglucose (FDG) accumulation in the lesion, with no suspicious findings of lymph node or distant metastases (Fig. 1b, c). He was diagnosed with resectable pancreatic tail cancer. Neoadjuvant chemotherapy was not administered, and the patient underwent an up-front surgery—distal pancreatectomy with D2 lymph node dissection. Intraoperative para-aortic lymph node sampling was negative. The postoperative course was eventful with the occurrence of grade B pancreatic fistula, which was conservatively treated with drainage and resolved. Pathological diagnosis was invasive ductal carcinoma of the pancreas, pT3N2M0 pStageIII (TNM Classification of Malignant Tumours, UICC 8th edition). S-1 adjuvant chemotherapy was initiated. Three months postoperatively, the levels of tumor markers—carcinoembryonic antigen (CEA), CA19-9, DUPAN-2, and SPan-1 in peripheral blood were 5.1 ng/mL (< 5.0 ng/mL), 619 U/mL (< 37 U/mL), 1100 U/mL (≤ 150 U/mL), and 160 U/mL (≤ 30 U/mL), respectively. Moreover, contrast-enhanced CT and FDG-PET/CT revealed local recurrence in para-aortic lymph nodes (Fig. 1d). Chemotherapy was revised to a combined regimen of gemcitabine and nab-paclitaxel. After 4 cycles of chemotherapy, all tumor markers were within the normal limits. After 5 cycles, local recurrence could not be identified on CT. Complete remission was achieved, and chemotherapy was administered for a total of 29 cycles with a reduction in dose. After the 29th cycle, the patient presented with complaints of sudden headache and vomiting. CT revealed metastatic brain tumors (Fig. 2). Two lesions were identified on magnetic resonance imaging (MRI): a 20-mm lesion in the left frontal lobe and a 32-mm lesion in the left cerebellum (Fig. 3). No other metastases were identified. The patient had symptoms of cerebral hypertension and underwent emergency craniotomy. Tumor markers, which were examined 1 month prior to surgery, showed no significant elevation. However, on the day of surgery, CA19-9 level was elevated (86 U/mL). Conversely, SPan-1 and CEA levels were slightly elevated (34 U/mL and 5.8 ng/mL, respectively). Intraoperative cerebrospinal fluid (CSF) samples showed prominent elevation of CEA (89 ng/mL), CA19-9 (1442 U/mL), DUPAN-2 (220 U/mL), and SPan-1 (390 U/mL). The neurological symptoms rapidly improved following the complete resection of the metastatic brain tumors, and no remnants were observed on imaging (Fig. 4). Pre-operative FDG-PET/CT was not performed because emergency surgical intervention was required to resolve the symptoms of cerebral hypertension; however, FDG-PET/CT performed on postoperative day 7 showed no findings of FDG accumulation, including in the para-aortic lymph nodes, which were suspected to be a recurrence (Fig. 1e). The patient was discharged on postoperative day 12, without any complications. A total of 53 Gy of local radiation was performed to suppress local recurrence. The pathological examination results of the brain and primary tumor specimens are shown in Fig. 5. Immunohistochemical analysis revealed that the neoplasm cells were positive for CK7 and CK19, and negative for S100 protein, similar to the primary tumor. Additionally, the metastatic brain tumors were positive for CDX2 and the Mib-1 index increased from 50 to 70%. However, the other histological and immunohistochemical features of the metastatic brain tumors were diverse from the primary tumor. In addition, no other primary tumors were present, including in the gastrointestinal tract.

In order to determine the effective chemotherapy regimen, cancer gene panel tests for the primary and metastatic tumors were performed. The results of the mutant genes without variant of uncertain significance are shown in Table 1. Tumor mutation burden increased from 6 Muts/Mb in the primary tumor to 8.7 Muts/Mb in the metastatic tumor. Variant of uncertain significance increased from 8 to 15. In addition, mutation of *CDKN2A/B*, *MTAP*, and *TP53* was found in the primary tumor and not in the metastatic brain tumors. However, mutation of *KRAS* and *BRCA2* was found in both primary and metastatic tumors. Additionally, the variant allele frequency of *KRAS* in the metastatic brain tumors was markedly increased.

**Table 1:** Mutated genes in primary tumor and brain metastases of pancreatic cancer

Sample	Gene	Mutation	Variant allele frequency (%)
Primary	*BRCA2*	S1341fs*33	46.4
	*KRAS*	G12C	11.16
	*CDKN2A/B*	Loss	**–**
	*MTAP*	Loss	**–**
	*TP53*	Y220N	5.82
Metastases	*BRCA2*	S1341fs*33	47.9
	*KRAS*	G12C	41.8

On postoperative day 30, blood CEA had decreased to 5.4 ng/dL, and CA19-9, CA125, DUPAN-2, SPan-1, and elastase 1 (≤ 300 ng/dL) were all within the standard range. A lumbar puncture was performed for the purpose of reassessing tumor markers in CSF and for cytological diagnosis. Tumor markers in CSF were CEA (< 0.5 ng/mL), CA19-9 (< 2.0 U/mL), DUPAN-2 (≤ 25 U/mL), and SPan-1 (2.5 U/mL). Cytology of the CSF revealed no tumor cells. Six months postoperatively, there has been no recurrence of the metastatic brain tumors.

## Discussion

The incidence of pancreatic cancer brain metastasis is reported to be 0.33–0.6% [1, 2]. Therefore, CT and MRI of the brain are rarely performed during routine follow-up of pancreatic cancer in clinical practice. Elevated tumor markers can be a strong indicator of tumor recurrence; however, in the present case, a significant increase in the levels of tumor markers did not occur until the symptoms of raised intracranial pressure had developed. This phenomenon might be attributed to the blood–brain barrier (BBB). The BBB, which separates the central nervous system from the rest of the body, restricts the transfer of substances, including chemotherapy drugs, into the brain; only small, lipid-soluble molecules with a molecular weight < 400 Da cross the BBB [4]. Therefore, the presence of neurological symptoms is a more clinically significant sign of brain metastasis than tumor marker levels because the transfer of tumor markers across the BBB might have been restricted.

There are several reports of brain metastases of pancreatic cancer even after effective chemotherapy (G-FLIP, FOLFIRINOX, gemcitabine plus nab-paclitaxel) [3, 5, 6] similar to the present case. These reports suggest the limited efficacy of existing chemotherapy regimens. Gene panel test of the resected tissue has been performed for identifying specialized anti-cancer drugs. Different mutant genes have been identified in the primary and metastatic brain tumors. It has been reported that the mutations are not similar in the metastatic and primary tumors of lung cancer [7]; however, it is possible that a similar situation may be observed in pancreatic cancer, although the cancers are different. *KRAS* and *BRCA2* mutations were found in both primary and metastatic tumors. *KRAS* mutations are observed in 94% and *BRCA2* mutations in 6% of patients with pancreatic cancer [8]. Olaparib is indicated for patients with *BRCA2* mutations responding to platinum-based therapy. However, the central transferability of olaparib is low; therefore, the effect is considered to be limited.

According to the clinical practice guidelines of European Association of Neuro-Oncology and European Society for Medical Oncology, surgery should be considered when the patient has acute symptoms caused by raised intracranial pressure [9].

Additional treatment might be needed to achieve radical cure even in cases where metastatic brain tumors can be resected. Patients with pancreatic cancer metastases to other organs have a poor prognosis. However, some recent reports have shown that patients with solitary brain metastasis had acquired a good long-term prognosis after resection of the metastatic tumor. Table 2 summarizes the reports of brain metastases in patients with pancreatic cancer who acquired complete resection to primary and metastases [10–12]. Most of the metastases were isochronous and single. Two cases, including our present case, have described the presence of unelevated tumor markers in peripheral blood. The time of postoperative brain metastases diagnosis varies from 1 to 6 years; hence, even a patient with a survival time of more than 5 years may develop solitary brain metastasis.

**Table 2:** Cases of brain metastases of pancreatic cancer with other recurrences under control

Author	Age/sex	BM pattern/number	Peripheral blood TM	Time from PC surgery to BM	BM treatment	PC treatment	BM recurrence	Survival after BM treatment
Caricato et al. [10]	67/male	Heterochrony/1	Normal range	2 years	Surgery	Surgery CRT	Yes, 16 months after surgery	> 16 months
Lemke et al. [11]	48/female	Heterochrony/1	None	6 years	Surgery WBRT (46 Gy)	Surgery CT	No	> 10 years
	66/male	Heterochrony/1	None	Approximately 1 year	Surgery WBRT (30 Gy)	Surgery CT	No	> 5 years
Chiang et al. [12]	54/male	Simultaneity/1	None	–	Surgery WBRT (45 Gy)	Surgery CRT	No	> 20 months
Our case	64/male	Heterochrony/2	Normal range	2 years and 5 months	Surgery SRT (53 Gy)	Surgery CT	No	> 6 months

*BM* brain metastasis, *TM* tumor marker, *RT* radiation therapy, *PC* pancreatic cancer, *CRT* chemoradiation therapy, *CT* chemotherapy, *WBRT* whole-brain radiotherapy, *SRT* stereotactic radiotherapy

All patients underwent resection of the metastatic brain tumors. In the present case, there was a marked decrease of tumor markers in the CSF after aggressive surgical resection. The serum/CSF ratios of CEA and CA19-9 in healthy subjects have been reported, with median values of 7.800 and 9.867, respectively [13]. So, the reference range for CEA and CA19-9 in CSF are < 0.641 ng/mL and < 3.750 U/mL, respectively; in the present case, CEA and CA19-9 in CSF were within the reference range after resection of metastatic brain tumors. Therefore, it was adjudged that the tumors were completely resected. Four patients underwent additional radiotherapy; one patient who did not receive radiotherapy later had a recurrence of brain metastases. Better local control has been reported in metastatic brain tumors with the addition of postoperative irradiation compared with that achieved through surgical resection alone [14]. Therefore, postoperative irradiation was performed in the present case. Patients who underwent resection of metastatic brain tumors were treated with whole-brain irradiation (WBR). However, side effects, including progressive dementia, ataxia, and urinary incontinence have been reported [15]. Recently, it has been reported that salvage stereotactic radiosurgery (SR) was non-inferior to WBR [16]. Hence, we decided to add salvage SR in the present case. Therefore, aggressive surgical resection and salvage SR could lead to increased local control of metastatic tumor and a more favorable prognosis. Six months postoperatively, the patient has had no recurrence.

## Conclusion

Brain metastases of pancreatic cancer are rare. Since only a limited number of drugs can cross the BBB and enter the central nervous system, resection is a viable option for a small number of resectable brain metastases. Aggressive resection and salvage SR may lead to an R0 state and have a good long-term prognosis.

## Data Availability

Not applicable.

## Abbreviations

CT: Computed tomography
PET: Positron emission tomography
FDG: Fluorodeoxyglucose
CEA: Carcinoembryonic antigen
MRI: Magnetic resonance imaging
BBB: Blood–brain barrier
WBR: Whole-brain irradiation
SR: Stereotactic radiosurgery
